# MicroRNA-137 has a suppressive role in liver cancer via targeting EZH2

**DOI:** 10.3892/mmr.2022.12661

**Published:** 2022-03-01

**Authors:** Shichang Cui, Yanlei Sun, Yang Liu, Chengbiao Liu, Jinbao Wang, Guang Hao, Qidong Sun

Mol Med Rep 16: 9494–9502, 2017; DOI: 10.3892/mmr.2017.7828

Following the publication of this paper, it was drawn to the Editors’ attention by a concerned reader that the Transwell cell migration assay data shown in Fig. 2D and 6D, and the scratch-wound assay data in Figs. 2E and 6E, were strikingly similar to data appearing in different form in other articles by different authors. Owing to the fact that the contentious data in the above article had already been published elsewhere, or were already under consideration for publication, prior to its submission to *Molecular Medicine Reports*, the Editor has decided that this paper should be retracted from the Journal. The authors were asked for an explanation to account for these concerns, but the Editorial Office did not receive any reply. The Editor apologizes to the readership for any inconvenience caused.

